# A housekeeping gene search to analyze expression changes of individual genes in Macaca mulatta

**DOI:** 10.18699/vjgb-25-138

**Published:** 2025-12

**Authors:** M.V. Shulskaya, A.Kh. Alieva, I.R. Kumakov, M.I. Shadrina, P.A. Slominsky

**Affiliations:** National Research Center “Kurchatov Institute”, Moscow, Russia; National Research Center “Kurchatov Institute”, Moscow, Russia; National Research Center “Kurchatov Institute”, Moscow, Russia; National Research Center “Kurchatov Institute”, Moscow, Russia; National Research Center “Kurchatov Institute”, Moscow, Russia

**Keywords:** Macaca mulatta, expression analysis, “housekeeping gene”, real-time PCR, expression, Macaca mulatta, экспрессионный анализ, «ген домашнего хозяйства», ПЦР в реальном времени, экспрессия

## Abstract

Rhesus macaques (Macaca mulatta) are the most common non-human primates living in captivity. The use of rhesus macaques as model objects is determined, first of all, by their phylogenetic and physiological closeness to humans, and, as a consequence, the possibility of extrapolating the obtained results to humans. Currently, it is known that a number of biochemical changes occur under various physiological conditions, including at the transcriptomic level. The real-time polymerase chain reaction is a widely used universal method for gene expression analysis. Carrying out such studies always requires a preliminary selection of “housekeeping genes” (HKGs) – genes necessary for the implementation of basic functions in the cell and stably expressed in different cell types and under different conditions. At present, there are only two systematic studies on the search for HKGs in the rhesus macaque brain, and therefore in this work a search and systematization of HKGs for this species were carried out. As a result, two panels of promising HKGs for M. mulatta were formed: an extended panel, consisting of 56 genes, and a small panel, consisting of 8 genes: ARHGDIA, CYB5R1, NDUFA7, RRAGA, TTC1, UBA6, VPS72, and YWHAH. Both panels of potential HKGs do not have pseudogenes in macaques or humans, are characterized by stable and sufficient expression in the brain of rhesus macaques and can be used to analyze expression not only in the brain but also in peripheral blood. However, it should be noted that the data have not been experimentally verified and require verification in laboratory conditions.

## Abstract (ru)

Макаки резус (Macaca mulatta) являются наиболее распространенными нечеловекообразными приматами, их используют в качестве модельных объектов, в первую очередь, из-за филогенетической и физиологической близости к человеку. В настоящее время модельные организмы широко используются для целого ряда исследований, в том числе на уровне транскриптома. При этом для анализа экспрессии отдельных генов применяется универсальный метод – полимеразная цепная реакция в реальном времени. Проведение такого рода исследований всегда требует предварительного подбора «генов домашнего хозяйства» (ГДХ) – генов, необходимых для реализации основных функций в клетке и стабильно экспрессирующихся в различных типах клеток и при разных условиях. На сегодняшний день для макак резус существуют лишь две систематизированные работы по поиску ГДХ, однако эти исследования проводились лишь для тканей мозга и не учитывают такой важный критерий, как связь ГДХ с заболеваниями. В связи с этим в нашей работе были сформулированы ключевые критерии, учитываемые при подборе ГДХ. Проведены поиск и систематизация кандидатных ГДХ. В результате сформированы две панели перспективных ГДХ для M. mulatta: расширенная панель на 56 генов и малая панель, состоящая из восьми генов: ARHGDIA, CYB5R1, NDUFA7, RRAGA, TTC1, UBA6, VPS72 и YWHAH. Обе панели соответствуют всем критериям подбора ГДХ (не имеют псевдогенов ни у макаки, ни у человека, характеризуются стабильной и достаточной экспрессией в мозге макак резус и могут быть использованы для анализа экспрессии не только в мозге, но и в периферической крови). Однако необходимо отметить, что данные экспериментально не верифицированы и требуют проверки в лабораторных условиях.

## Introduction

Rhesus macaques (Macaca mulatta) have served as a model for studying various human diseases for decades. Their use as a model is primarily explained by the phylogenetic and physiological similarity to humans, and, consequently, the potential for transferring the results obtained. To date, genetic models of cancer (Brammer et al., 2018; Deycmar et al., 2023), cardiovascular diseases (Patterson et al., 2002; Ueda et al., 2019), ophthalmologic diseases (Singh et al., 2009; Liu et al., 2015; Moshiri et al., 2019; Peterson et al., 2019, 2023), skeletal diseases (Colman, 2018; Paschalis et al., 2019), diseases of the reproductive system (Lomniczi et al., 2012; Nair et al., 2016; Abbott et al., 2019), as well as a wide range of neurological diseases (McBride et al., 2018; Sherman et al., 2021) are known in rhesus macaques. In addition, rhesus macaques are used for research as model objects of toxicity (Kaya et al., 2023), radiation (Li et al., 2021; Majewski et al., 2021), hormones (Noriega et al., 2010; Eghlidi, Urbanski, 2015), etc. In addition to studying diseases, this model can be used to test various pharmacological drugs, which is especially important for applied research.

It is now known that a wide range of biochemical changes occur under various physiological conditions, including at the transcriptome level. Relative transcript levels of individual genes can be accurately and reproducibly measured using real-time polymerase chain reaction (RT-PCR). This method is a widely used and versatile tool for analyzing the expression of a small number of genes. RT-PCR is also frequently used to confirm results obtained using whole-transcriptome expression analysis (Ramsköld et al., 2009). However, this type of study is always complicated by variations in the copy number of the target mRNA due to differences in the amount of total RNA between samples, therefore requiring the preliminary selection of control (reference) genes, or “housekeeping genes” (HKGs).

The term HKG most often refers to genes stably expressed in various cell types and under various conditions and required for basic cellular functions. They are often used as reference genes in gene expression studies to normalize mRNA levels between different samples.

In rhesus macaques, there is currently very little systematic data on the use of HKGs (Ahn et al., 2008; Noriega et al., 2010). Noriega et al. (2010) conducted a study only on the brain, while Ahn et al. (2008) worked with both brain tissue and some other tissues (intestine, liver, kidney, lung, and stomach). However, neither of these studies examined the animals’ peripheral blood, which is widely used for various expression studies. In this regard, this review conducted a search and systematization of data on HKGs in rhesus macaques for their further use in studying gene expression changes under various conditions.

## Modern principles of selection of HKGs

Currently, the selection of HKGs is based on the following main principles. First, the absence of pseudogenes, copies of genes that contain certain defects in the coding region (loss of introns and exons, frameshifts, or premature stop codons, as well as pseudogenes formed as a result of retrotransposition), is an important criterion for selecting HKGs (Tutar, 2012). Pseudogenes are not involved in protein processing but can be expressed at the RNA level. Furthermore, the number of pseudogenes is known to be unstable in the genomes of different individuals. From a practical standpoint, the presence of pseudogenes may require additional treatment of the analyzed RNA samples with DNases, which is critical for samples with low RNA amounts. Therefore, the presence of pseudogenes is highly undesirable when selecting HKGs.

Second, expression stability is considered to be another important criterion for selecting HKGs, i. e., they should have relatively constant expression levels across different cell types, tissues, and experimental conditions (Tu et al., 2006). However, it is known that HKGs can be expressed differentially in different tissues. For example, well-known HKGs such as beta-actin and GAPDH have been shown to vary significantly in expression levels across tissues (Cai J. et al., 2014). Therefore, a high level of HKGs’ expression in the specific tissue under study is an important criterion.

Third, there is increasing support for the idea that HKGs should be tailored to specific experimental conditions (Silver et al., 2008). For example, the human HSPA8 gene is a HKG, but it cannot be used as such in the study of age-related or neurodegenerative diseases, as there is evidence of a decrease in HSPA8 gene expression with age, as well as an association between this gene and the development of neurodegenerative diseases (Loeffler et al., 2016; Tanaka et al., 2024). Expression profile variability has also been demonstrated for HKGs used in the study of cancer (de Kok et al., 2005; Dheda et al., 2005). To date, no studies have identified all-purpose HKGs, meaning that HKGs’ selection for the specific pathology being studied is necessary

**Fig. 1.**
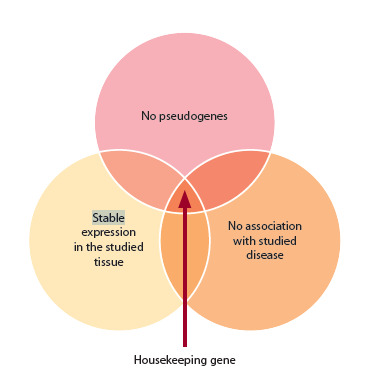
Main HKG criteria

Thus, an ideal HKG should have no pseudogenes, no association with the disease or condition being studied, and it should be stably expressed under specific experimental conditions and tissues (Fig. 1). The optimal HKG should be carefully selected for each specific experiment. Using multiple HKGs also improves the reliability of the expression data obtained (Vandesompele et al., 2002; Dheda et al., 2005).

## Analysis of the published data on HKGs in rhesus macaques

We screened scientific publications in the PubMed database to find papers focused on the analysis of HKGs in rhesus macaques. An initial search using the keywords (gene expression) AND (rhesus macaque) identified 3,017 publications. Since “rhesus macaque” and “Macaca mulatta” are synonymous, both terms were used in the analysis of search queries. Due to the relatively large number of publications returned, the search query was specified using the synonymous terms “housekeeping genes” and “reference genes”, which yielded 126 and 97 search results, respectively. Further narrowing the search by refining it using the keyword “rt-pcr” revealed 16 and 7 publications (Table 1).

**Table 1.**
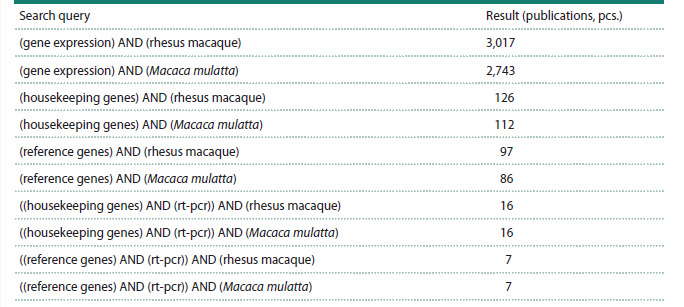
Names of search queries in the PubMed database (https://pubmed.ncbi.nlm.nih.gov/) Notе. Accessed on April 28, 2025.

A detailed analysis of these seven studies identified two most relevant systematic studies to date on the selection of HKGs in rhesus macaques (Ahn et al., 2008; Noriega et al., 2010). Five of the seven remaining publications analyzed did not mention HKGs and were therefore not included in the analysis

Next, a block of 126 open-access publications found in PubMed using the keywords (housekeeping genes) AND (rhesus macaque) was manually analyzed. It was found that 107 publications, for one reason or another, did not mention any HKGs, while 16 publications used genes recommended by the authors of the two main studies on the selection of HKGs in rhesus macaques (Ahn et al., 2008; Noriega et al., 2010). These two types of publications were excluded from further analysis. Our search yielded only one additional publication (Robinson et al., 2018). Supplementary Table S11 summarizes the data from these three key studies and describes 115 genes expressed in the rhesus macaque brain that could be considered as HKGs. These genes were selected for further analysis.

Supplementary Materials are available in the online version of the paper: https://vavilov.elpub.ru/jour/manager/files/Suppl_Shulskaya_Engl_29_8.pdf


Due to periodic database updates, some gene names were updated and given with names different from those used in (Ahn et al., 2008; Noriega et al., 2010) when compiling this list. Four sequences that were homologous to human sequences but were absent in the Ensembl database for rhesus macaques (Genome assembly: Mmul_10 (GCA_003339765.3)) (Table S1) and five M. mulatta genes currently identified as having pseudogenes (LDHB, RPL37, RPS27A, SNRPA, and SUI1) were also excluded.

This procedure allows us to identify all-purpose HKGs for both humans and macaques, while also avoiding problems associated with the low level of annotation of the rhesus macaque genome assembly. For example, the RPL19 gene, currently the most widely used HKG in rhesus macaques, is not recommended for use as an all-purpose HKG because it has pseudogenes in human genome

The genes selected after the previous screening steps can be used for studies on brain tissue. However, peripheral blood, widely used in human studies, is of particular interest. Peripheral blood is promising for expression studies due to its availability and low invasiveness. Therefore, we considered it necessary to select candidate HKGs for peripheral blood, for the purpose of which the selected genes were further analyzed for acceptable expression levels in peripheral blood (Table S2).

Since peripheral blood expression data are currently completely lacking for M. mulatta, and due to the similarity between the macaque and human transcriptomes, publicly available mRNA expression data were analyzed in human whole blood and lymphoblasts. We also included expression data in mice, as these animals are a well-studied model object (due to the lack of peripheral blood data, tissues with similar expression patterns, such as bone marrow, lymph nodes, and spleen, were used). Expression data in the brain and spleen of rhesus macaques were added from the Ensembl database (Table S2).

This analysis was conducted using the BioGPS database (http://biogps.org/), where we selected genes with expression above the median in the tissues of interest. “Median expression” represents the 50th percentile of the expression data, meaning that half of the tissues have expression levels below the median, and the other half have expression levels above the median. BioGPS uses this metric to provide a summary of how a gene is expressed in different tissues, conditions, or data sets.

**Fig. 2.**
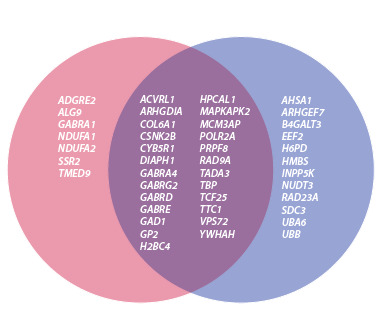
xpression of candidate HKGs in selected human and mouse tissues. Genes expressed predominantly in humans are shown in pink, and genes expressed predominantly in mice are shown in purple. The overlapping area indicates genes expressed in specific tissues of both species.

As a result of the analysis, the list of genes was divided into three groups: genes with expression levels above the median in both humans and mice, genes with expression levels above the median in only one of the two species, and genes with expression levels below the median in both humans and mice (Fig. 2, Table S2). Genes from all three groups can be considered as candidate HKGs. However, their use will limit the number of model objects compared based on their expression profiles. Genes from the first group are the most promising. It should also be noted that the expression data presented in BioGPS require experimental verification in the laboratory.

**Fig. 3.**
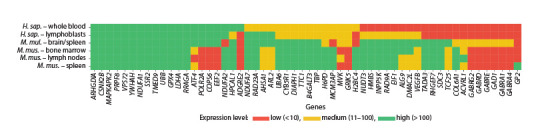
Heatmap of relative expression levels of candidate HKGs. Median-normalized values for each gene in the BioGPS resource (http://biogps.org) were used as the basis.

However, it is important to note that the median value is not always a good indicator for selecting candidate genes, since the mRNA abundance in the tissue under study may be higher than the median, but the absolute expression levels are quite low. Therefore, all analyzed genes were ranked according to their relative expression levels in the analyzed tissues. The results of this analysis are presented as a heat map (Fig. 3). Ultimately, we formed a group of 25 most promising candidate HKGs (genes with high or moderate expression levels in humans, mice, and rhesus macaques).

Since HKGs can be used to study changes in the expression of various genes in various diseases, potential HKGs should not be implicated in the development of the disease under study. A selected group of 25 genes was analyzed using the MalaCards database (www.malacards.org). MalaCards is a searchable, integrated knowledge base containing comprehensive information on human diseases, medical conditions, and disorders. We searched for associations between the gene and currently known disease models in rhesus macaques (Table 2). Six genes associated with oncological diseases (AHSA1, B4GALT3, HPCAL1, TBP, TMED9, and SSR2), six genes associated with neurological diseases (CSNK2B, DIAPH1, MAPKAPK2, NDUFA1, RAD23A, and UBB), as well as genes associated with eye diseases (ARL2 and PRPF8) and some other diseases (GPX4 and LDHA) were excluded.

**Table 2.**
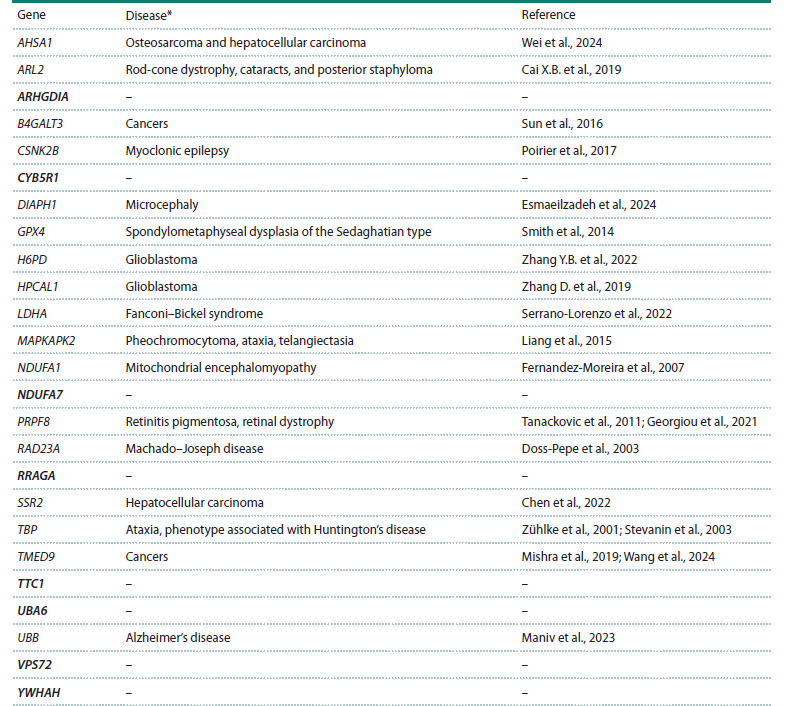
Association of the selected highly expressed potential HKGs with disease groups modeled in rhesus macaques Pubmed (https://pubmed.ncbi.nlm.nih.gov/) has no published data for 2000–2025.

As a result, at this final stage of the selection of candidate HKGs, we selected eight genes (ARHGDIA, CYB5R1, NDUFA7, RRAGA, TTC1, UBA6, VPS72, and YWHAH – highlighted bold in Table 2), characterized by the absence of pseudogenes, the absence of data on the involvement of these genes in the development of diseases modeled in rhesus macaques, as well as stable and high expression in the analyzed tissues (brain, peripheral blood, spleen, lymph nodes, bone marrow).

## Conclusion

Thus, two panels of promising HKGs for M. mulatta were formed: an extended panel consisting of 56 genes (Table S2) and a small panel consisting of 8 genes (Table 2). Both panels of potential HKGs have no pseudogenes either in macaques or in humans, and they are characterized by stable and sufficient expression in the rhesus macaque brain. However, the specialized panel is more all-purpose, as it is suitable for selecting HKGs for parallel studies on several model organisms (mouse, macaque, and human) or for studying several different diseases simultaneously by a single research group. The small panel is of interest for further development of a working HKGs panel to study changes in the expression of various genes in various diseases in M. mulatta. At the same time, the extended panel of potential HKGs is also quite promising.

## Conflict of interest

The authors declare no conflict of interest.
